# DOES MOVING LATER IN LIFE INFLUENCE IN-PERSON CONTACT WITH CHILDREN, FAMILY, AND FRIENDS?

**DOI:** 10.1093/geroni/igz038.630

**Published:** 2019-11-08

**Authors:** Joonyoung Cho, Jacqui Smith

**Affiliations:** University of Michigan, Ann Arbor, Michigan, United States

## Abstract

Relocation is common in later life and older adults differ the distance they move. One possible consequence of relocation is that in-person contact frequency with social network members changes. To date, relatively little is known about how older adults’ in-person contact frequency with their children, family members, and friends is influenced by the distance they move and if this differs by age group (50-64, 65-74, 75+). To examine this, we used information from the Health and Retirement Study about geographic mobility and social network contact frequency. The sample was restricted to respondents over age 50 in the 2006 wave with data on contact frequency with children, family members, and friends in 2006 and 2014 (N=5159). Distance moved from 2006 to 2014 was categorized as: stayer, <5 miles, 5–49.9 miles, and ≥50 miles. Linear regressions with covariates controlled revealed that moving ≥50 miles was significantly associated with less frequent in-person contact with family members (β=-0.31, 95%CI [- 0.46, -0.16], p≤0.001) and friends (β =-0.32, 95%CI [-0.46, -0.18], p≤0.001). Interestingly, the 50-64 group who moved ≥50 miles was less likely to meet up with their children (β=-.0.36, 95%CI [-0.56, -0.15], p≤0.001), family members (β=-0.31, 95%CI [-0.52, -0.11], p≤0.01) and friends (β=-0.21, 95%CI [-0.39, -0.03], p ≤ 0.05). In contrast, the 75+ group who moved ≥50 miles were significantly less likely to have in-person frequency only with their friends (β=-0.58, 95%CI [-1.04, -0.14], p≤0.05). These findings are discussed in relation to theories about social support and emotional well-being in old age.

